# U-Compare bio-event meta-service: compatible BioNLP event extraction services

**DOI:** 10.1186/1471-2105-12-481

**Published:** 2011-12-18

**Authors:** Yoshinobu Kano, Jari Björne, Filip Ginter, Tapio Salakoski, Ekaterina Buyko, Udo Hahn, K Bretonnel Cohen, Karin Verspoor, Christophe Roeder, Lawrence E Hunter, Halil Kilicoglu, Sabine Bergler, Sofie Van Landeghem, Thomas Van Parys, Yves Van de Peer, Makoto Miwa, Sophia Ananiadou, Mariana Neves, Alberto Pascual-Montano, Arzucan Özgür, Dragomir R Radev, Sebastian Riedel, Rune Sætre, Hong-Woo Chun, Jin-Dong Kim, Sampo Pyysalo, Tomoko Ohta, Jun'ichi Tsujii

**Affiliations:** 1 Precursory Research for Embryonic Science and Technology (PRESTO), Japan Science and Technology Agency (JST), 4-1-8 Honcho, Kawaguchi, Saitama, 332-0012, Japan; 2 National Institute of Informatics (NII), 2-1-2 Hitotsubashi, Chiyoda-ku, Tokyo, 101-8430, Japan; 3 Department of Information Technology, University of Turku, Joukahaisenkatu 3-5, Turku, FI-20014, Finland; 4 Turku Centre for Computer Science (TUCS), Joukahaisenkatu 3-5 B, Turku, FI-20520, Finland; 5 Jena University Language & Information Engineering (JULIE) Lab, Friedrich-Schiller-Universität Jena, Fürstengraben 30, D-07743 Jena, Germany; 6 Computational Bioscience Program, University of Colorado Denver School of Medicine, PO Box 6511, MS 8303, Aurora, CO 80045 USA; 7 Department of Computer Science and Software Engineering, Concordia University, 1455 de Maisonneuve Blvd West, Montréal, QC, H3G 1M8, Canada; 8 Department of Plant Systems Biology, VIB/Ghent University, Technologiepark 927, Ghent, B-9052, Belgium; 9 The National Centre for Text Mining (NaCTeM) and School of Computer Science, the University of Manchester, Manchester, UK; 10 National Center of Biotechnology-CSIC, C/Darwin 3, Campus de Cantoblanco, Madrid, 28049, Spain; 11 Department of Electrical Engineering and Computer Science, University of Michigan, Ann Arbor, MI 48109, USA; 12 Department of Computer Engineering, Bogazici University, 34342 Bebek, Istanbul, Turkey; 13 School of Information, University of Michigan, Ann Arbor, MI 48109, USA; 14 Department of Computer Science, University of Massachusetts Amherst, Massachusetts, USA; 15 Department of Computer and Information Science (IDI), Norwegian University of Science and Technology (NTNU), Sem Sælands vei 7-9, Trondheim, NO-7491, Norway; 16 Korea Institute of Science and Technology Information. 245 Daehangno, Yuseong-gu, Daejeon, 305-806, South Korea; 17 Database Center for Life Science (DBCLS), Research Organization of Information and System, Faculty of Engineering Bldg. 12, the University of Tokyo, 2-11-16, Yayoi, Bunkyo-ku, Tokyo, 113-0032 Japan; 18 Department of Computer Science, University of Tokyo, Tokyo, Japan; 19 Microsoft Research Asia, Building 2, No. 5 Dan Ling Street, Haidian District, Beijing, 100080 P.R.China

## Abstract

### Background

Bio-molecular event extraction from literature is recognized as an important task of bio text mining and, as such, many relevant systems have been developed and made available during the last decade. While such systems provide useful services individually, there is a need for a meta-service to enable comparison and ensemble of such services, offering optimal solutions for various purposes.

### Results

We have integrated nine event extraction systems in the U-Compare framework, making them inter-compatible and interoperable with other U-Compare components. The U-Compare event meta-service provides various meta-level features for comparison and ensemble of multiple event extraction systems. Experimental results show that the performance improvements achieved by the ensemble are significant.

### Conclusions

While individual event extraction systems themselves provide useful features for bio text mining, the U-Compare meta-service is expected to improve the accessibility to the individual systems, and to enable meta-level uses over multiple event extraction systems such as comparison and ensemble.

## Background

Since the release of event-annotated corpora [1,2], and due to the BioNLP shared task in 2009 [3] and 2011 [4], many event extraction tools for biological literature have become publicly available. While such tools provide useful functionalities individually, there are several obstacles hindering non-expert users from finding and utilizing the best tools for their specific challenges. First, such tools are not easy to use especially when they need to be customized, e.g. when used with a particular named entity recognizer. Second, individual tools are developed with different user interfaces, and it is often time-consuming to get accustomed with the various usages of tools, especially when multiple systems need to be tested for e.g. comparison. Thus, the interoperability and accessibility are crucial issues to improve the usability.

A similar case can be found with the BioCreative challenge [5,6] and MetaServer [7]. BioCreative has been particularly concerned with extracting protein-protein interactions (PPIs). In the BioCreative II.5 challenge [5,6], participants provided PPI extraction tools as web services through the BioCreative MetaServer [7]. Providing a unified interface to the input and output of the various PPI extraction tools, the BioCreative MetaServer enabled easy access to those tools, and showed the necessity of a meta-level service of information systems. In the BioNLP '09 shared task on event extraction [3], participants presented tools which extract biological events with richer and more fine grained information than the BioCreative challenges. However, the shared task required static files of processed data on a given corpus; event extraction tools themselves were not available. To resolve this issue, our event extraction meta-service now provides interactive event extraction services in the fine grained BioNLP shared task style.

Roughly speaking, the goal of the BioNLP shared task is to extract biological events from literature, given their raw text and protein annotations. The BioNLP shared task defines "txt", "a1" and "a2" formats for this event extraction task. A "txt" format file contains raw text of a biomedical paper, while the corresponding "a1" format file includes protein named entity boundaries annotated on that paper. Participants of the shared task were required to submit "a2" format files, which define extracted events and may refer to protein annotations in the corresponding "a1" files. In the shared task evaluation, submitted "a2" files were compared with the gold standard "a2" files which were manually annotated by human curators.

Our services are interoperable with other UIMA/U-Compare services, which allow users to create customized workflows easily. UIMA, Unstructured Information Management Architecture, is an interoperability framework for unstructured information in general. UIMA is provided as an Apache open source project and is widely used in the NLP domain. A UIMA component can either be a local service or a web service, and both types can be freely mixed to create a UIMA workflow.

U-Compare provides a broad range of UIMA compliant components including BioNLP components such as protein taggers and annotated corpus readers. Compatibility of these components is guaranteed by sharing data type definitions. U-Compare also provides a UIMA compliant integrated NLP platform. The U-Compare platform provides direct access to the U-Compare components, where local components are automatically downloaded and executed on demand. A local component has the advantage of portability although users are required to install the original tool in case of a non-Java implementation. On the other hand, a web service component can have limitations in its computational capacities. The U-Compare platform allows easy workflow creation from these components or any third party UIMA components. Additionally, U-Compare provides a comparison and evaluation feature implemented in a UIMA compliant way [8]. U-Compare shows the results of workflow runs both statistically and visually. All of these features are available without any programming necessity.

We have integrated the bio-event meta-service, which we describe in this paper, to the U-Compare platform. This integration could accelerate developments of text mining in the bioinformatics area. The most straightforward usage of our system would be to combine a few text mining tools and run the resulting pipeline on any text relevant to a specific biological use-case. Our system makes such a usage dramatically easier compared with the existing systems.

While our ready-to-use services themselves are very useful especially for the end users of text mining, comparison of the various bio-event services is critical when the users need to develop a state-of-the-art application. For example, developers need deeper analysis of the behaviours of the event extraction systems in order to select the most suitable service among available services. However, even the original service developers do not know the behaviour of their services, because those services are black-box and different text input would cause unknown behaviours. Therefore, users need to analyse comparisons of the service outputs by inputting text for a specific domain of interest. Our system is the first system to allow such a comparison of the event extraction services that output complex event structures. Our comparison system does not just calculate statistical scores but also helps users to analyse the comparisons by visualization features.

Furthermore, ensemble of the services has large potential to improve the individual performance. It is known in general that an ensemble of the text mining services could improve the performance significantly. Our system allows the creation of such an ensemble for end users.

All of the above use cases require the meta-service, which can provide compatible, interoperable, and ready-to-use bio-event services. As our system supports such usages, users can create text mining applications for their individual purposes in an efficient and effective way.

## Results

We describe below possible use cases of our event extraction services. We assume that users would first create a workflow and run it, and subsequently analyse the result to find a relevant workflow for their use-case.

### Workflow composition

**Figure 1:**
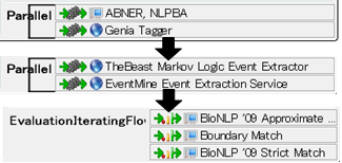
**A conceptual figure of a combinatorial comparison example**. Two protein taggers and two event extractors make four combinations, which will be compared by three metrics.

Text processing workflows involving event extraction can be composed using a wide variety of NLP tools readily available in the U-Compare repository. For example, Figure 1 shows two protein taggers and two event extraction components among others from the repository. Given them, users can compose four workflows by simply combining them. In the U-Compare repository, there are currently four protein taggers and nine event extraction services. A workflow created in U-Compare can be saved in a local storage and launched by executing the Java command below:

$ java -Djavaws.workflow.path="path/workflow.xml" -cp . UCLoader -jnlp http://u-compare.org/lib/u-compare-runworkflow.jnlp

which makes integration of the workflow into a user system straightforward.

### Performance comparison

**Figure 2:**
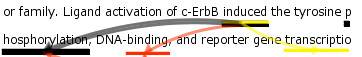
**A screenshot visualizing comparison between event extraction tool A and B**. Events of tool A colored in red, events of tool B colored in yellow, and matched events highlighted in black.

The performance of the workflows can be evaluated in various ways. For example, Figure 1 shows three quantitative evaluation methods available from U-Compare. Users can also evaluate the performance by directly reviewing the results (Figure 2). Table 1 shows evaluation scores of our event extraction services using the test set of the BioNLP '09 gold standard corpus. Because implementation of the evaluation metric used in this paper is slightly different from the one used in the BioNLP '09 shared task, the evaluation scores are also different. The bottommost row of Table 1 shows the original F1 scores in the BioNLP '09 shared task while some of the services have updated their systems showing better performance. We will make the source code of the evaluation metric publicly available.

**Table 1:** Evaluation scores of event extraction services.

Participant		JULIE Lab JReX	UTurku	EventMine	BExtract	VIBGhent	TheBeast	UMich	Moara	CCP- BTMG
Rank in F1 score	#	1	2	3	4	5	6	7	8	9

Total	F1	51.09	49.91	48.20	44.48	42.44	37.19	36.34	29.50	22.03
	
	PR	57.69	56.32	64.00	61.56	59.05	48.15	35.57	31.99	70.03
	
	RC	45.85	44.81	38.65	34.82	33.12	30.30	37.15	27.31	13.07

Localization	F1	61.60	55.85	63.20	51.45	51.79	48.98	53.47	44.19	17.80

Binding	F1	49.24	45.43	39.86	26.97	34.42	34.50	31.75	28.36	20.92

Gene expression	F1	72.48	71.67	72.63	65.14	69.57	59.28	66.00	58.79	51.07

Transcription	F1	42.99	50.21	50.00	24.71	57.14	17.48	30.06	26.40	22.93

Protein catabolism	F1	80.00	50.00	60.87	60.00	68.97	72.00	58.06	50.00	40.00

Phosphorylation	F1	81.99	79.70	81.29	80.69	76.23	72.79	77.15	52.88	33.33

Regulation	F1	31.20	33.97	28.77	32.21	19.39	29.96	14.29	10.83	5.79

Positive Regulation	F1	40.39	38.66	28.25	35.83	23.34	29.57	21.50	14.68	6.69

Negative Regulation	F1	38.47	36.28	32.62	33.27	26.67	27.32	26.61	13.16	4.01

BioNLP '09 ST Total Evaluation	F1	46.66	51.95	36.88	44.62	40.54	44.35	19.28	24.15	22.66

Rows show scores in total and scores for each event types.

### Ensemble result of event extraction services

Table 2 shows the result of the ensemble. While the best F1 score of the original event extraction services was 51.09, an averaged voting of the top 5 services performed 56.91. This 5.82 point increase in F1 score can be regarded as a significant improvement. The scores in Table 2 were obtained by selecting the best one among different threshold settings on the same test set data. For this reason, it would not be fair to directly compare these results with the original event extraction tools which are tuned on the development set data; instead this result does show the potential of an ensemble of event extraction tools.

**Table 2:** Result of the ensemble.

Ensemble	F1	PR	RC
Top 2	52.06	48.88	55.69

Top 3	53.80	73.21	42.52

Top 4	56.44	70.38	47.11

Top 5	56.91	67.17	49.37

Top 6	56.64	63.39	51.19

Top 7	55.21	57.60	53.02

Top 8	54.87	68.31	45.85

Top 9	54.81	67.57	46.10

F1, PR, RC stand for F1 score, precision and recall respectively. Evaluation was performed by the BioNLP shared task's approximate matching metric.

Although using top × services showed a single peak in their score curve in this case, the F-scores may not necessarily include sufficient information when selecting which services to include in the ensemble. For example, [CCP-BTMG] and [BExtract] are rule based systems while others are machine learning based systems. Thus [CCP-BTMG] and [BExtract] could potentially contribute to the ensemble performance even if their total evaluation scores were lower. Such a ranking which considers uniqueness of services rather than simply ranking by F-score is regarded as interesting future work.

## Conclusions

We provide state-of-the-art services in an interoperable and ready-to-use way, which allows users to easily find and run customized workflows suitable to their purposes. Even though our event extraction services were originally created towards the same shared task goal, each service has different characteristics. Furthermore, each service may behave differently depending on its input. We showed that ensemble of event extraction services performs better than the original services as we can exploit these differences between the individual services.

As more event extraction tools will be published in the future, adding more compatible services is regarded as interesting future work. Processing larger data sets would also be a future work.

## Methods

We provide nine state-of-the-art event extraction services, originally developed for the BioNLP '09 shared task. The required input of services is arbitrary text, with annotated protein named entity boundaries. Then all of the services have been made interoperable with U-Compare [9] which is an integrated NLP system based on the UIMA framework [10]. Exploiting the interoperability and the compatibility of these services, we performed ensemble, comparison and evaluation of these services. In this section we describe the details of our event extraction services and the ensemble methods.

### Event extraction services

In this subsection, we describe the details of each event extraction service we provide.

#### Concordia University BExtract event extraction system [BExtract]

BExtract [11] is a linguistically inspired, rule-based system for biological event extraction. It relies on a dictionary of categorized trigger expressions to detect and characterize biological event expressions and syntactic dependency based heuristics to extract their event arguments. Trigger expressions are drawn from the BioNLP'09 Shared Task training data, and are further refined based on several constraints: only verbs, nouns, and adjectives are considered as triggers, each trigger is limited to one event class, and unlikely triggers are filtered out by thresholding. Event argument identification is based on finding dependency paths between trigger expressions and named entities (or other triggers) in the sentence, dependency relations along the path determining the semantic role of the argument. The relevant dependency paths have been automatically induced from the training data and finalized via manual filtering. Two grammatical constructions, coordination and apposition, are given special attention at this stage. Several simple post-processing rules deal with the limitations of the approach to ensure increased recall. The final system consists of 325 trigger expressions and 33 event argument identification rules. The BExtract system also addresses speculation and negation detection in similar manner, the only difference being that the trigger expressions were manually compiled for this task. The system was ranked 3^rd ^in core event extraction task in the Shared Task (2^nd ^in extraction of complex regulatory events) and 1^st ^in speculation and negation detection task.

#### VIB/Ghent University event extraction system [VIBGhent]

The VIBGhent extraction system [12] consists of a fully parallelized supervised learning framework that first extracts a set of physical events (i.e. Localization, Binding, Gene expression, Transcription, Protein catabolism and Phosphorylation) before retrieving more complex and nested regulatory events (i.e. Regulation, Positive regulation and Negative regulation). A separate SVM classifier is trained in parallel for each of the different event types, enabling the system to learn type-specific features. A rich feature set is automatically generated from the training data, incorporating lexical features from the sentence and grammatical/syntactic features of the shortest path in the dependency graph. One additional strength of the system is its ability to classify one trigger into different event types; e.g. the word "overexpression" that can lead to both a Gene expression as well as a Positive regulation event. A weaker point however is the error-propagation of predicting physical events to the prediction of regulatory events. As a result, the overall system achieved a 5th place in the official Shared Task 2009 evaluation (40.54% F-score), while the evaluation of only the physical events reveals a third place (57.85% F-score). Our system has been improved further for inclusion in the Bio-Event Meta-Service.

#### National Center of Biotechnology Moara event extraction system [Moara]

The Moara [13] event extraction system uses a two-step case-based reasoning approach. The first step is the multi-class named-entity extraction system for the identification of the trigger events and the site and location entities. The case is represented as a window of tokens of [-1,+1] with the following features: lemma of the token, part-of- speech tag, distance to the closest protein (number of tokens, in multiples of five), direction to the closest protein (right or left), distance in terms of dependency tag (in multiples of two), type of event together with the BIEWO tag and the modifier of the event (negation, speculation or none). The second step is the relationship extractor and the cases are a representation of the local context using the following features: concatenated string of the entities and roles of the context (e.g., "Site:Entity, Theme:Protein, Trigger:Binding"), part-of-speech tag, type of entity (e.g., Protein, Binding, Entity, etc.), role (e.g., Theme, Cause, etc.) and lemma for each token in the context. In both steps, the system search for the most similar cases and the final solution is given by a majority voting among the selected cases.

#### EventMine [EventMine]

EventMine [14] is a machine learning based pipeline system for event extraction, following the strategy by the UTurku system [15]. EventMine has been developed from scratch after the shared task. EventMine consists of three modules: a trigger detector, an event edge detector, and an event detector. The trigger detector classifies each word into the appropriate event types, the event edge detector classifies each edge between an event and a candidate participant into an argument type, and the event detector classifies event candidates constructed by all edge combinations, deciding between event and non-event. The former two modules solve multi-label classification problems, and the latter one solves multi-class classification problems, with one-vs-all support vector machines. EventMine is fully machine learning based, and the strength of EventMine is the configurability and the adaptability. EventMine can employ multiple outputs of the parsers as additional features. EventMine can be configured to other problem settings, and is easily adaptable to other corpora by retraining the system. EventMine has been developed by Tsujii Laboratory, the University of Tokyo, and subsequently by the National Centre for Text Mining (NaCTeM), University of Manchester.

#### thebeast event extraction system [thebeast]

Instead of dividing the event extraction process into a cascade of trigger and argument extraction, the [thebeast] event extraction system [16] uses a *joint *statistical model of both triggers and arguments. In this model information can flow backwards: evidence for arguments can directly help trigger extraction. Joint Modelling has been successfully applied to many NLP problems. Notably, the highest ranking systems [17,18] for several of the 2011 shared task are based on a joint model inspired by the work of [16]. The statistical model applied in this work is specified through Markov Logic, a combination of Markov Networks and First Order Logic. Using a set of soft constraints ("the word inhibit implies a Negative_Regulation event") and hard constraints ("events must have at least one theme"), a global model of events is defined. Inference and Learning in this model is performed using thebeast - a Markov Logic engine tailored for NLP problems. As learning scheme MIRA online learning is used, for inference CPI [19].

#### University of Michigan event extraction system [UMich]

The UMich event extraction system [20] is based on supervised machine learning with features extracted from the dependency parse trees of the sentences. After segmenting the text into sentences by using MxTerminator, we use the Stanford Parser [21] to generate the dependency parse trees of the sentences. The candidate triggers are detected by using a dictionary based approach, where the dictionary is extracted automatically from the BioNLP'09 shared task training and development data sets. Noisy trigger candidates such as "with", "+", ":", and "-", which are rarely used as real triggers and commonly used in other contexts, are filtered out. The event types are grouped into three general classes based on the number and types of participants that they involve. The first class includes the event types that are described with a single theme participant. The second class includes the event types that are described with one or more theme participants. The third class includes the events that are described with a theme and/or a cause participant. Separate support vector machine (SVM) models are learned for each class of events to classify each candidate event trigger/participant pair as a real trigger/participant pair or not. An edit-distance based kernel function is defined on the dependency relation paths between the candidate trigger/participant pairs and integrated to SVM. Although the official F-score performance of the system was 19.28% at the shared task due to a bug in our software, the fixed system achieved an F-score of 39.83% on the same data set.

#### The University of Turku Event Extraction System [UTurku]

The Turku Event Extraction System [15] is a pipeline event extraction system that uses a unified, extensible graph representation, where protein entities and event triggers are the nodes and event arguments the edges. The system uses SVMs to first predict event trigger nodes, followed by prediction of event argument edges. The resulting graph is "pulled apart" into individual events by a rule-based unmerging component. These steps can be followed by post-processing, such as prediction of speculation and negation (BioNLP Shared Task task 3) or conversion to the Shared Task file format.

The Turku system relies heavily on syntactic dependency parses, represented as graphs of token nodes and dependency edges, linked to the event graph through matching entity/token pairs. The parse is the main source of features for the SVM classification steps. In particular, the features of the edge detector are largely based on the *shortest connecting path of dependencies *between the two entity nodes of an edge.

The Turku system had the best performance in the BioNLP'09 Shared Task with 51.95% F-score. The version integrated into U-Compare is based on the improved system that achieved a performance of 52.86% [22].

#### JULIE Lab JReX [JULIE Lab JReX]

JULIE LAB JREX (Jena Relation eXtractor) is an event and relation extraction system developed by the Jena University Language & Information Engineering (JULIE) Lab. It originated from JULIE Lab's involvement in the BioNLP 2009 Shared Task on Event Extraction (cf. [23] for a deeper account), and was continuously improved after the competition [24]. The event extraction pipeline of JREX consists of two major parts, the pre-processor and the dedicated event extractor. The JREX pre-processor uses a series of JCORE text analytics tools [25] such as sentence splitter, tokenizer, POS tagger, chunker, and named entity recognizer (among them GENO, a high-performance gene/protein name tagger and normalizer; cf. [26]). JREX heavily exploits the syntactic structure of sentences in terms of dependency trees. For dependency parsing, the JREX pre-processor actually comes with the MST parser [27], retrained on the GENIA Treebank version 1.0 [28]. The second main component of JREX, the event extractor, accounts for three crucial subtasks - first, the detection of lexicalized event triggers, second, the trimming of dependency graphs which involves eliminating informationally irrelevant lexical material from the dependency parse tree and enriching informationally relevant lexical material by conceptual labels on increasing levels of conceptual abstration, and, third, the identification and ordering of arguments for the event under scrutiny. The JREX event extractor is composed of manually curated dictionaries to annotate potential event triggers, rules for dependency tree trimming procedures, and machine learning technology to sort out associated event triggers and arguments on trimmed dependency graph structures. The JReX version provided in U-Compare achieves a performance of 45.9% recall, 57.7% precision and 51.1% F1-score on the BioNLP'09 Shared Task test data [24].

#### UC Denver, Computational Bioscience Program [CCP-BTMG]

The foundation of the CCP event mining system is the OpenDMAP [29] semantic parser and a set of manually conceived rules. For this challenge, the OpenDMAP concept recognition system was augmented with a broad ontology defined for the events of interest, new linguistic patterns for those events, and specialized coordination handling. The overall system uses a pipeline approach facilitated by the use of UIMA. The system uses a combination of tools for named entity recognition of the semantic classes, including the LingPipe GENIA tagging module and several dictionary lookup components, all based on the UIMA-sandbox ConceptMapper tool. The coordination module uses the OpenNLP constituent parser. From the constituent parse, coordination structures are extracted into a simplified data structure that captures each conjunction along with its conjuncts. The coordination analysis is used in particular to identify events in which the THEME argument was expressed as a conjoined noun phrase. OpenDMAP patterns are designed to take advantage of the high quality ontologies available in the biomedical domain. They aim to model how concepts can be expressed in text taking advantage of both semantic and linguistic characteristics of the text. Patterns were manually constructed for each event type by examining the training data and by using native speaker intuitions about likely ways of expressing relationships, similar to the technique described in [30]. The patterns characterize the linguistic expression of that event and identify the arguments (participants) of the events according to (a) occurrence in a relevant linguistic context and (b) satisfaction of appropriate semantic constraints, as defined by our ontology. Pattern matches are scored using a weighted average of three component scores. The first is a simple scoring algorithm for ranking competing matches beginning at a given token in the text. This algorithm prefers matches that cover every word of the span to those that have intervening words. The second is a pattern score that penalizes matches to patterns which have optional pattern elements that are uninstantiated in the match. The third is a concept score that penalizes matches that do not fill all slots associated with a concept in the ontology are also incorporated into the final pattern match score. The result of this approach is a very high precision information extraction system that has limited recall due to the coverage of the pattern set. The system achieved state-of-the-art precision in both the core event extraction (71.81) and event enrichment (70.97) tasks.

### U-Compare Bio-Event Meta-Service

We built the U-Compare bio-event meta-service which helps developers to easily deploy their event extraction services as a U-Compare compatible UIMA component. Each developer is simply required to prepare an event extraction tool, which accepts only raw text and protein boundary information as input, without any other preprocessing. The U-Compare bio-event meta-service has an open architecture where individual event extraction services can easily be registered to interoperate with other U-Compare components. An original event extraction tool should be first wrapped into a UIMA/U-Compare local service component. Then this local UIMA component can be deployed as a web service. Finally the new event extraction component, either local or web service, can be registered to the repository. We created a service development package which helps developing and deploying services with minimal human effort of the developers. This package provides a wrapper which converts BioNLP shared task format files (a1 file defines protein boundaries and a2 file contains event annotations) to/from UIMA data structure using U-Compare compatible type definitions. Developers then simply need to prepare a command line tool which receives the locations of the input text file and the input a1 file. Subsequently, the tool outputs the a2 file to a specified location. By specifying the tool's command path, our wrapper works as a local UIMA component. Deploying as a web service is also easily available by setting a few parameters such as a service port number. Either local or web service deployments are ready-to-run as generic UIMA components at this stage. While providing a service is an open procedure independent from the U-Compare platform, we integrated the event extraction services into the U-Compare platform. This integration allows users to skip an explicit installation process. We can integrate a new service as users require.

### Ensemble of event extraction services

In this subsection, we first describe the comparison and evaluation methods. Then we describe the ensemble methods which further build upon the result of comparison and evaluation.

**Figure 3:**
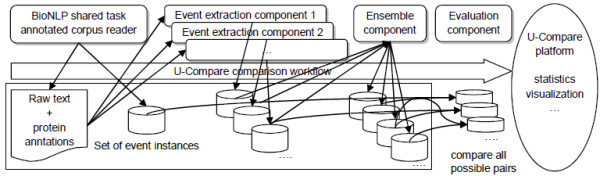
**A conceptual figure of the ensemble architecture**. A workflow which reads a gold standard corpus, runs a couple of event extraction tools, and aggregates their results.

We created a BioNLP '09 style evaluation component which works together with the combinatorial comparison feature of the U-Compare platform. Figure 3 illustrates the architecture of this comparison and evaluation. The BioNLP '09 evaluation component supports a strict matching metric and an approximate matching metric, both for each event type as well as globally. We only use the approximate matching metric in this paper. The evaluation component calculates F1, precision and recall statistics for all of the possible pairs of components specified in the workflow. The evaluation component also holds information of matched and unmatched event instances, which is used in further analyses such as visualization. If one of the event extraction components is compared with our BioNLP corpus reader component, this comparison is regarded as an evaluation of that event extraction component using the BioNLP shared task's gold standard corpus. Otherwise, the comparison result shows the similarity between two event extraction components. Table 1 depicts the evaluation scores on the test set of the BioNLP '09 gold standard corpus for each event extraction service. The test set gold standard data is not publicly available for the developers to fairly compare their system performance.

As we created nine compatible event extraction components, the ensemble of these components would be a natural extension. We performed ensemble annotation by (weighted) majority voting. One issue when calculating votes is that we do not know which instances of extracted events can be considered as "equal" to sum up the voting counts. We implemented our evaluation component in a generic way, which can determine an equality of two arbitrary event instances using the evaluation metrics of the BioNLP shared task.

The next issue is vote weighting. Some of the event extraction components may perform better than others, or may output useful results which others cannot. Thus weighting votes depending on the characteristics of each event extraction component and would improve the final result. We performed a couple of different weighted voting experiments.

An averaged ensemble is a voting method where all of the annotations have an equal weight. For each extracted event, the voting threshold condition of an averaged ensemble is as follows:

$$\begin{array}{c} \underset{i}{\sum }b_{i}>th\mathrm{re}shold,\\ b_{i}:=\left\{\begin{array}{c} 1\;if\;equal\;event\;exists\;in\;i_{th}\;service\\ 0 \end{array}\right) \end{array}$$

while an optimal threshold value is unknown. Changing a threshold of a majority voting normally shows a trade-off between precision and recall. Hereafter we show the results of a single threshold value which is optimized to obtain the best F-score.

Using all of the available event extraction components may result in lower scores even if we assign weights to votes, because the characteristics of the components cannot be expressed in a single scalar value of weights. We tried using the top n (n = 2, 3, ..., 9) components to observe the influence of different component combinations on the final ensemble performance. This top n rank was decided by the F-scores evaluated by the test set of the gold standard corpus shown in Table 1.

Ensembles were performed by a U-Compare comparison workflow using UIMA components we created for this task. The workflow uses the following components: the BioNLP shared task reader as a collection reader; then a parallel aggregate which children are the shared task user data readers, each of them reading one individual participant's result; and finally the special ensemble component.

## Authors' contributions

YK: meta-service system design and implementation, ensemble related works. JDK, SP, TO, YK and JT: BioNLP '09 shared task organization. All others: event extraction services and their deployments. Author names were sorted by alphabetical order of last names but authors of same research team are grouped together, except for the shared task organizers. All authors have read and approved the final manuscript.
